# Development of Plant-Based Adipose Tissue Analogs: Freeze-Thaw and Cooking Stability of High Internal Phase Emulsions and Gelled Emulsions

**DOI:** 10.3390/foods11243996

**Published:** 2022-12-09

**Authors:** Xiaoyan Hu, David Julian McClements

**Affiliations:** Department of Food Science, University of Massachusetts, Amherst, MA 01003, USA

**Keywords:** plant-based food, adipose tissue, soy protein, coconut oil, temperature stability

## Abstract

There is great interest in the development of plant-based alternatives to meat products to meet the rising demand from vegans, vegetarians, and flexitarians. Ideally, these products should look, feel, taste, and behave like the meat products they are designed to replace. In this study, we investigated the impact of simulated freeze–thaw and cooking treatments on the properties of plant-based adipose tissues formulated using high internal phase emulsions (HIPEs) or gelled emulsions (GEs). The HIPEs consisted of 75% oil, 2% soybean protein, 23% water, while the GEs consisted of 60% oil, 2% soybean protein, 2% agar and 36% of water. Low melting point (soybean oil) and high melting point (coconut oil) oils were used to create emulsions with either liquid or partially crystalline lipid phases at ambient temperature, respectively. In general, GEs were harder than HIPEs, and emulsions containing coconut oil were harder than those containing soybean oil at ambient temperatures. The thermal behavior of the plant-based adipose tissue was compared to that of beef adipose tissue. Beef adipose tissue was an opaque whitish semi-solid at ambient temperature. These properties could be mimicked with all types of HIPEs and GEs. The structure of the beef adipose tissue was resistant to freezing/thawing (−20/+20 °C) but not cooking (90 °C, 30 min). Soybean HIPEs and GEs were relatively stable to simulated cooking but not freeze–thawing. Conversely, coconut HIPEs and GEs exhibited the opposite behavior. These results have important implications for the formulation of alternatives to animal adipose tissue in plant-based foods.

## 1. Introduction

There is growing interest in the creation of next-generation plant-based foods, like meat, seafood, egg, and milk analogs, that accurately simulate the look, feel, taste, and behavior of the animal-based foods they are designed to replace [1,2,3]. This trend is mainly driven by environmental, health, and ethical concerns linked to livestock production and the meat industry [4,5]. Despite the rapid growth in the development of meat analogs over the past few years, they still make up less than around 1% of the total retail market for meat and meat-like products in the United States [6]. Greater consumer adoption of these products in the future will depend on improvements in their quality attributes, nutritional profiles, and affordability.

One of the main reasons given for the poor acceptance of plant-based meats by consumers is that they do not accurately mimic the desirable organoleptic attributes of animal meat, such as their appearance, texture, mouthfeel, and flavor [7]. Animal meat has a characteristic tenderness, juiciness, and flavor, which is partially determined by the type and amount of fat it contains [8]. The fats in edible animal tissues are usually located within adipose tissue [9]. The main structural elements in adipose tissue are adipocytes, which are fat cells surrounded by membranes containing phospholipids and proteins [10]. These adipocytes are usually around 100 μm in diameter, have a polyhedral shape, and are embedded in collagenous networks [11]. In animals, the adipocytes can be located inside (intramuscular) or outside (intermuscular) the muscle tissues [12,13]. The adipocytes play an important role in determining the desirable quality attributes of meat products and so it is important to mimic their physicochemical and functional attributes when designing plant-based meat analogs.

Emulsion technology is particularly suitable for creating adipose tissue analogs for plant-based food applications [14,15]. Emulsions are colloidal systems consisting of two or more immiscible liquids, where at least one of the liquids is dispersed as small droplets in another [16]. Oil-in-water (O/W) emulsions, which consist of oil droplets dispersed in water, can be designed to have structures and properties analogous to those of animal adipose tissue. The individual oil droplets consist of a fat-rich core surrounded by a layer of amphiphilic molecules, which can be designed to resemble the cell membranes of adipocytes. The oil droplet concentration can be manipulated to reach the high fat levels found in adipose tissue. Moreover, plant-based additives can be included in the aqueous phase of O/W emulsions to simulate the behavior of collagen and other substances in the intercellular matrix. In this study, we examined the suitability of two kinds of emulsion technologies to mimic the properties of beef adipose tissue: high internal phase emulsions (HIPEs) and gelled emulsions (GEs).

**Figure 1:**
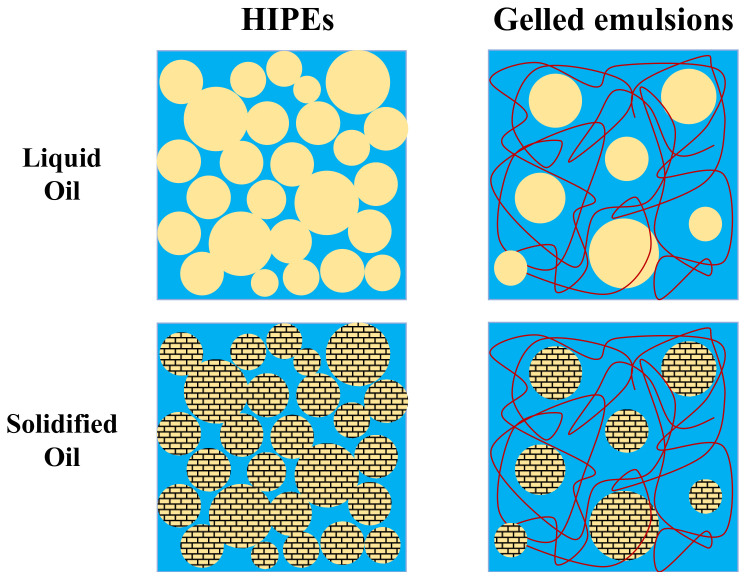
Structural differences between HIPEs and gelled emulsions containing either liquid oil or partially solidified solid fat.

HIPEs are emulsions where the dispersed phase concentration is so high (>74% *v*/*v*) that the droplets are jammed together, which leads to solid-like characteristics [17,18]. In the absence of pigments, HIPEs are optically opaque semi-solids that mimic some of the characteristics of animal adipose tissue [15]. However, they are typically too soft at ambient temperature and do not exhibit the characteristic softening of real adipose tissue when heated above body temperature [14,15]. These limitations may be overcome by utilizing high-melting fats that crystallize around body temperature, rather than low-melting ones that are liquid at this temperature (Figure 1). The formation of a fat crystal network within an emulsion increases its mechanical rigidity and provides the desired softening when heating. However, the presence of the fat crystals may also lead to instability of the emulsion due to partial coalescence [19]. Consequently, it is important to determine whether high-melting plant-based fats, like coconut oil, can be used to improve the functional performance of HIPEs designed for use as animal adipose tissue analogs.

Gelled emulsions can be created by adding hydrophilic gelling agents to the aqueous phase of O/W emulsions (Figure 1) [20,21]. The gelling agents can be designed to mimic the behavior of the collagen in animal adipose tissue. Collagen forms hydrogels at low temperature due to hydrogen bonding between helical regions [22]. Consequently, it may be possible to better simulate the behavior of real adipose tissue by using gelled emulsions that contain a plant-based cold-set biopolymer in their aqueous phase. Moreover, the presence of this biopolymer could improve the resistance of the emulsions to droplet aggregation and gravitational separation by slowing down their movement through the aqueous phase.

In this study, we investigated the possibility of creating adipose tissue analogs using HIPEs or gelled emulsions assembled from plant-based ingredients. The gelled emulsions contained agar, a cold-setting polysaccharide, to mimic the properties of collagen. The impact of using a high melting point (coconut oil) or low melting point (soybean oil) fat phase on the formation and functional performance of both adipose tissue analogs was also studied. We also examined the impact of simulated freezing/thawing and cooking on the stability and properties of the HIPEs and gelled emulsions because these are processing operations commonly used in the food industry. The knowledge obtained from this study may lead to the creation of plant-based meat analogs that better mimic the desirable physicochemical, functional, and sensory properties of animal meat.

## 2. Materials and Methods

### 2.1. Materials

Soy protein isolate was kindly provided by ADM (Decatur, IL, USA). Soybean oil (Wesson, Conagra, Chicago, IL, USA) and coconut oil (Nature’s Promise, Stop and Shop, Quincy, MA, USA) were purchased from a local grocery store. Agar was provided by TIC gums (Westchester, IL, USA). Bradford reagent was purchased from BIO-RAD (Hercules, CA, USA). Sodium azide, Nile red, and FITC were obtained from Sigma Aldrich (St. Louis, MO, USA). Sodium azide is a non-food grade preservative, which was used to inhibit microbial growth during storage of the samples. Sodium phosphate was purchased from Fisher Scientific (Waltham, MA, USA). Distilled and deionized water from a laboratory purification unit was used to prepare all aqueous solutions and emulsions.

### 2.2. Preparation of HIPEs

Sodium phosphate was dissolved in distilled and deionized water at a concentration of 10 mM and this buffer solution was then adjusted to pH 7.0 using concentrated NaOH or HCl solutions. Soy protein powder (15%) was dispersed in phosphate-buffered solution and then stirred at 4 °C over night to promote dissolution. The protein solution was then centrifuged in an ultracentrifuge (Sorvall LYNX 6000, Waltham, MA, USA) at 4 °C, 10,000 RCF for 30 min to remove any insoluble extraneous matter. The supernatant was collected and used as a protein stock solution in the remainder of the experiments. The protein concentration in the supernatant was established using the Bradford assay by measuring the absorbance of the protein solution at a wavelength of 595 nm using a UV-visible spectrometer. Sodium azide (0.02%) was added to the protein solutions to prevent microbial growth during storage (this preservative is not suitable for use in commercial food products).

Concentrated oil-in-water emulsions were prepared by blending 75% (*w*/*w*) oil phase and 25% (*w*/*w*) aqueous phase. Initially, an aqueous phase containing 2% soybean protein was prepared by diluting the protein stock protein solution with pH 7 buffer solution. Then, 20 g of total sample (oil, water, and protein) was weighed into a glass beaker (50 mL) and blended using a high-shear mixer (M133/1281-0, Biospec Products, Inc., ESGS, Switzerland) at 12,000 RCF for 1 min, following the method used in our previous study [14]. The soybean oil emulsions were prepared at ambient temperature, whereas the coconut oil emulsions were prepared at 40 °C to ensure the oil phase was completely melted. All samples were then stored at ambient temperature for at least 15 min prior to analysis.

### 2.3. Preparation of Gelled Emulsions

Initially, a stock agar solution was prepared. This was carried out by dispersing 2% (*w*/*w*) agar powder in buffer solution and then stirring for at least 4 h to disperse and hydrate the agar. This sample was then covered and heated to 90 °C for 30 min to fully dissolve the agar. The resulting solution was then cooled to ambient temperature by placing the container in water for 30 min, which led to the formation of a gel.

The gelled emulsions were prepared from an oil phase and agar solution containing soybean protein. Initially, a 5% (*w*/*w*) agar solution was prepared by dispersing powdered agar in buffer solution and then heating to 90 °C for 30 min. This solution was then mixed with stock soybean protein solution to form an aqueous phase containing 2% agar and 2% soybean protein in buffer solution. The aqueous phase and oil phase were then weighed into a 50 mL beaker, covered, and heated at 90 °C in a water bath. After 10 min incubation, the sample was blended using a high-shear mixer for 1 min at 12,000 rpm. To maintain the agar solution in a liquid state, the gelled emulsions were prepared at this high temperature. The resulting oil-in-water emulsions were then cooled down to ambient temperature by placing the container in a water bath for 30 min. The samples were then covered, removed from the water bath, and incubated for a further 15 min at ambient temperature before analysis. Gelled emulsions were prepared using either soybean oil or coconut oil as the oil phase using this method.

### 2.4. Impact of Heating and Freezing on Emulsion Properties

For beef adipose, HIPEs, and gelled emulsions, about 20 g of sample were placed in a clear falcon tube with a cap. For the heat treatments, the samples were heated for 30 min in a water bath at 90 °C. For the freeze–thaw treatments, the samples were placed in a refrigerator at −20 °C for 24 h and then heated to room temperature (around 20 °C) for 1 h.

### 2.5. Microstructure

The microstructure of the HIPEs and emulsion gels were examined using confocal scanning laser microscopy with a 10× eye lens and a 40× objective lens (Nikon Eclipse C1 80i, Nikon, Melville, NY, USA). The fluorescent oil and protein stains (Nile red and FITC) were dissolved in ethanol at 10 mg/mL, separately. A small amount of emulsion sample was then placed on a microscope slide and each dye was added. The sample was incubated with the dye solutions for around 30 s and then covered with a glass slip. Any excess dye solution was removed. Images were acquired and analyzed using the instrument’s software package (NIS-Elements, Nikon, Melville, NY, USA)

### 2.6. Rheology

The rheological properties of the samples were characterized using a dynamic shear rheometer (Kinexus, Malvern, Worcestershire, UK). Serrated parallel plates were used for all measurements to avoid slip effects. The diameter of the upper plate was 40 mm and the diameter of the lower plate was 65 mm. Initially, the instrument was calibrated to a 0 mm gap and then the test sample was loaded. The working gap was maintained at 1 mm for all rheological tests. Excess sample was removed from the measurement cell and the sample edges were covered with silicon oil to prevent evaporation during heating. Each sample was held in the cell for 10 min before analysis to ensure it reached the appropriate temperature.

The change in rheological properties were measured as a function of temperature at a fixed frequency (1 Hz) and strain (0.1%), which were in the linear viscoelastic regime (LVR). The samples were incubated at the initial temperature (0 °C) for 10 min, heated from 0 to 90 °C at 3 °C/min, held at 90 °C for 5 min, and then cooled from 90 to 0 °C at 3 °C per minute. The dynamic shear modulus was measured throughout this heat-cool cycle.

### 2.7. Differential Scanning Calorimetry Analysis

Any thermal transitions in the ingredients, HIPEs, or emulsion gels were detected using differential scanning calorimetry analysis (DSC 250, TA instruments, New Castle, DE, USA). Samples were weighed into high-volume aluminum pans and then sealed hermetically with a cap and seal ring. An empty pan was then used as a reference. Samples were initially equilibrated at 20 °C and then cooled from 20 to −40 °C at 5 °C/min, then heated from −40 to 100 °C, and then cooled down from 100 to −40 °C. Any phase transitions observed in the heat flow versus temperature profiles were then analyzed.

### 2.8. Texture Profile Analysis of Emulsions

Texture profile analysis of the emulsions was carried out using a texture analyzer (TA.XT Plus, Texture Technologies, Scarsdale, NY, USA). The instrument was calibrated using a 5000 g measurement cell. A 25 mm diameter flat cylinder probe was used to carry out the tests. The distance calibration was done by moving the upper probe plate from 0 to 40 mm relative to the lower platform. Test samples were prepared with 10 mm height and 30 mm diameter. A two-compression texture profile analysis (TPA) test was performed using a fixed final strain of 25% and a probe speed of 2 mm/s. After the first compression, the probe was held for 15 s before the second compression was executed. The hardness, adhesiveness, resilience, cohesion, and springiness of the samples were calculated from the force versus time curves.

### 2.9. Statistical Analysis

Each experiment was then carried out in triplicate with newly prepared samples and expressed as the mean and standard deviation. Significant differences were calculated by single-factor analysis of variance (one-way ANOVA) with post hoc Scheffe test using statistical software (SPSS).

## 3. Results and Discussion

### 3.1. Characterization of Beef Adipose Tissue

Initially, we characterize the properties of real beef adipose tissue so as to have a target when developing the plant-based adipose tissue.

#### 3.1.1. Appearance and Microstructure

**Figure 2:**
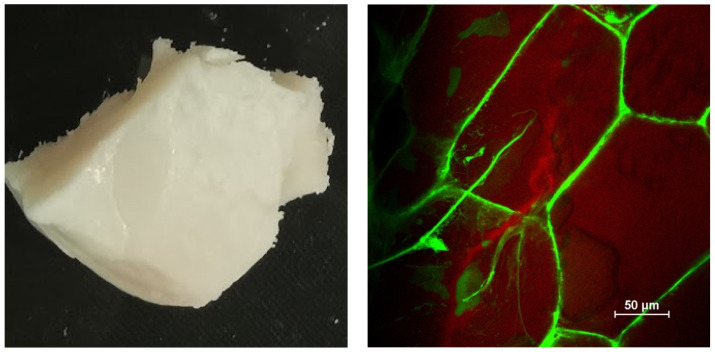
Appearance and microstructure of beef adipose tissue determined by digital photography and confocal fluorescence microscopy.

The appearance and misstructure of the beef adipose tissue was characterized using digital photography and confocal fluorescence microscopy (Figure 2). Visually, the beef adipose tissue was a white solid material with a slight yellowish tone. The opaque nature of the adipose tissue can mainly be attributed to light scattering by the fat crystals and adipose cells. The confocal fluorescence microscopy images indicated the beef adipose tissue consisted of lipid-rich polyhedral structures surrounded by protein-rich membranes. The average dimensions of the cells in the adipose tissue were around a 100 μm, which is consistent with previous studies [13].

#### 3.1.2. Temperature Dependence of Rheological Properties

**Figure 3:**
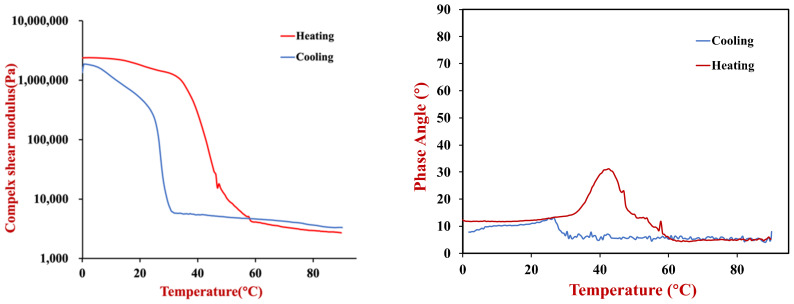
Temperature dependence of the complex shear modulus (**left**) and phase angle (**right**) of beef adipose tissue measured using dynamic shear rheometry (1 Hz, 0.1% strain).

The rheological properties of the beef adipose tissue was measured from 0 to 90 °C, which includes temperatures ranging from refrigeration to cooking [23]. Initially, we consider the changes in the rheological properties of the adipose tissue during heating. From 0–35 °C, the beef adipose tissue had a relatively high shear modulus, which can be attributed to the mechanical strength imparted by the crystal network formed by the solidified beef fat. From 35–60 °C, there was a steep decline in the shear modulus of the adipose tissue, which was attributed to melting of the beef fat and disruption of the fat crystal network. From 60–90 °C, there was only a slight reduction in the shear modulus with increasing temperature, which may be due to increased flexibility of the molecules. During heating, the phase angle was always below 45°, which indicated that the adipose tissue was always in a predominantly elastic state [24], even when the fat crystals had melted. This effect can be attributed to the presence of the protein-rich adipose cell walls (stained green in the microscopy images), which provided some mechanical rigidity because they do not breakdown during heating. Interestingly, there was a rise and then fall in the phase angle when the samples were heated from 35–60 °C (Figure 3). This effect was mainly attributed to the melting of the fat crystal network, as shown in the DSC results discussed later. The rheology of the adipose tissue was also measured when it was cooled from 90 to 0 °C. In this case, the shear modulus remained relatively low from 90 to 30 °C, and then increased sharpy, which was attributed to crystallization of the beef fat and the formation of a 3D crystal network that provided mechanical strength. The temperature at which the shear modulus increased sharply during cooling was lower than the temperature at which it decreased sharply during heating, which can be attributed to supercooling effects.

#### 3.1.3. Differential Scanning Calorimetry Analysis

**Figure 4:**
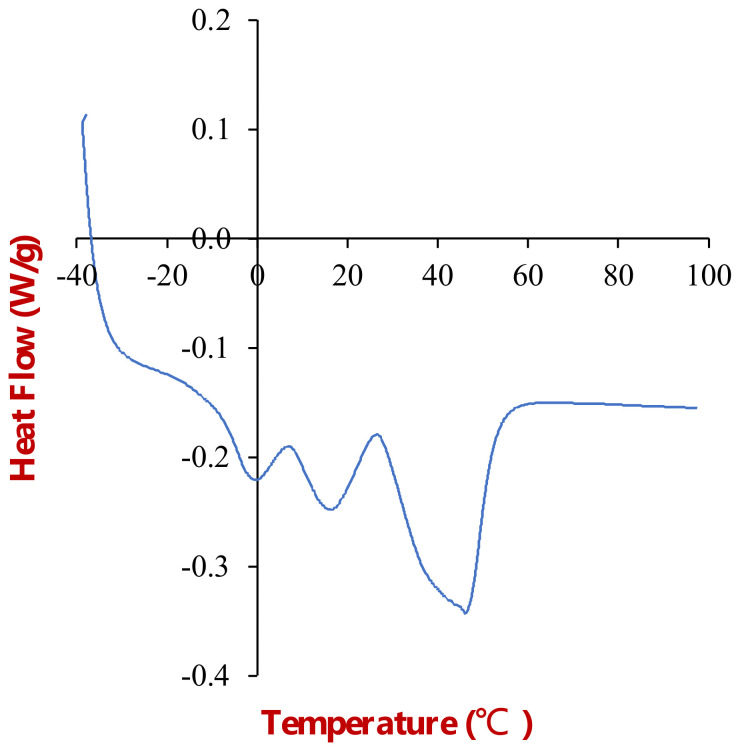
Differential calorimetry analysis of beef adipose tissue showing the change in heat flow with temperature when the sample was heated from −40 to 100 °C.

The thermal behavior of the beef adipose tissue was characterized by differential scanning calorimetry (Figure 4). The heat flow versus temperature profile of the samples was recorded from −40 to 100 °C. As found in our previous study, three thermal transitions were observed in the adipose tissue at 0, 15, and 45 °C [15]. The thermal transition at 0 °C was attributed to the melting of ice crystals formed when the adipose tissue was frozen, whereas the transitions observed at 15 and 45 °C were attributed to the melting of fat crystals. Notably, the fat phase did not fully melt until the temperature exceeded about 55 °C, which can be attributed to the high saturated fatty acid content of beef fat.

#### 3.1.4. Heating and Freezing Stability

**Figure 5:**
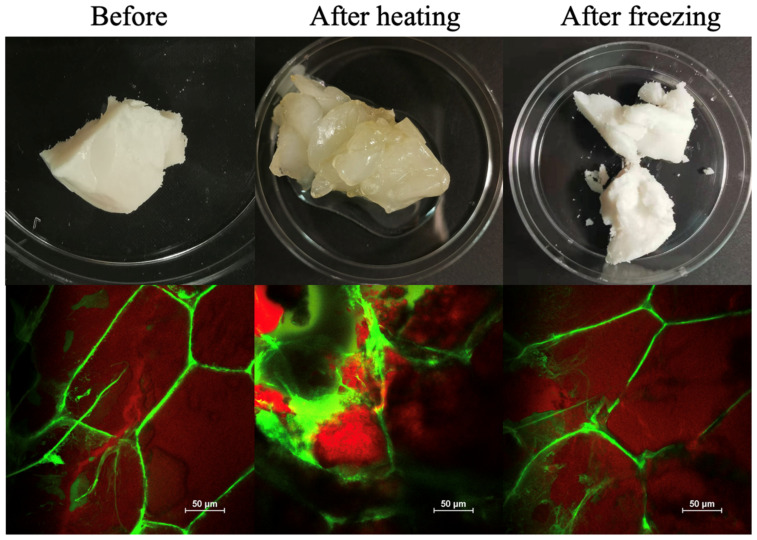
Appearance and microstructure of beef adipose tissue before and after heating and freezing for 24 h and then being brought to ambient temperature for 30 min before observation.

Animal meat is often frozen during storage and transport to extend its shelf life, and heated during food preparation to improve its palatability, desirability, and safety. Therefore, the resistance of beef adipose tissue to freeze–thawing and heating was determined (Figure 5).

There were clear changes in both the appearance and microstructure of the samples after heating and freezing.

Heating: Heating caused the fat phase inside the adipocytes to melt, which caused the appearance of the adipose tissue to change from opaque to semitransparent. However, the adipose tissue still remained solid, which can be attributed to the mechanical strength provided by the cell walls of the adipose tissue. Even so, the microscopy images show that there was some disruption of the adipose tissue structure after heating, which may have been due to the melting of the fat crystals and/or structural changes in the cell walls. For instance, collagen is known to denature around 65 °C, which would reduce the resistance of the intercellular matrix to disruption [25]. After the heated adipose tissue was cooled to ambient temperature, it became harder and opaquer, which can be attributed to the re-crystallization of the fat phase. Nevertheless, the original structure was not regained, indicating that some irreversible structural changes had occurred during the heating-cooling process. There appeared to be some leakage of fat from the samples after this process, which can be attributed to partial collapse of the adipose cell walls [26].

Freezing: The adipose tissue that had been frozen and thawed was still a semi-solid material, but it appeared to be whiter than the unfrozen sample (Figure 5). Freezing may have enhanced the formation of small fat crystals inside the adipocytes, which scattered light more strongly, thereby leading to an increase in lightness. Even so, there was little change in the overall microstructure of the beef adipose tissue after it was subjected to freezing and thawing. These results suggest that real beef adipose tissue has good freeze–thaw stability, which may be because the relatively high solids content of the aqueous phase between the adipocytes suppressed the formation of large ice crystals that could disrupt the structure.

### 3.2. HIPEs

We then characterized the thermal behavior of high internal phase emulsions formulated with different plant-based oils.

#### 3.2.1. Appearance and Microstructure

**Figure 6:**
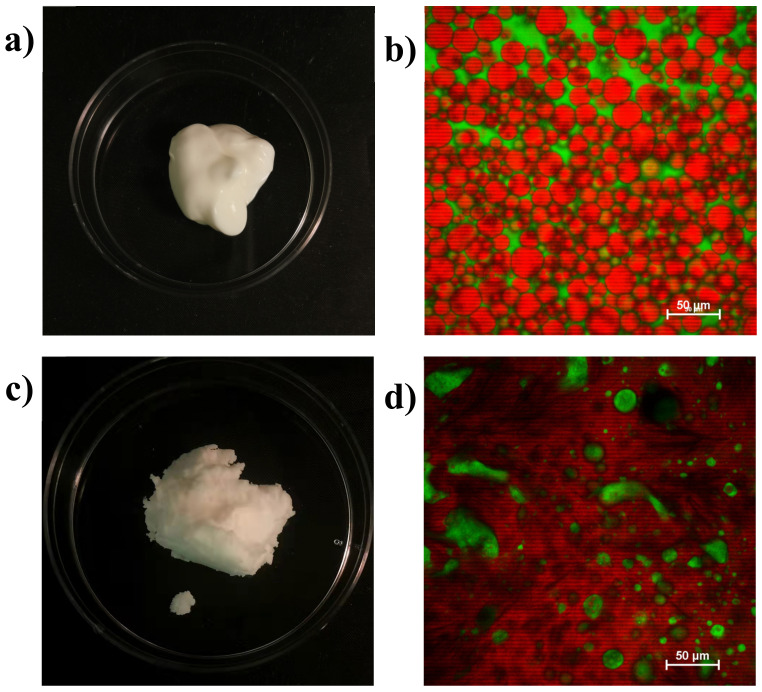
Appearance (**a**) and microstructure (**b**) of HIPEs containing soybean oil and appearance (**c**) and microstructure (**d**) of coconut oil characterized by digital photography and confocal fluorescence microscopy. The oil is stained red and the proteins green in the microscopy images.

HIPEs were prepared using soybean oil and coconut oil as the lipid phase to represent liquid oils and solid fats at room temperature, respectively. The appearance and microstructure of the HIPEs are shown in Figure 6.

Visually both HIPEs were solid-like and had a whitish color that was somewhat similar to that of real beef adipose tissue. However, the HIPEs formulated with soybean oil appeared softer and had a glossier surface than the ones formulated with coconut oil. This effect was mainly attributed to the formation of a fat crystal network within the coconut oil at room temperature, which provided some mechanical rigidity and light scattering. The smoother surfaces of the soybean HIPEs led to more specular reflectance, thereby giving a glossier appearance.

The confocal fluorescence microscopy images indicate that the soybean oil HIPEs were concentrated oil-in-water emulsions consisting of spherical oil droplets dispersed in a protein-rich aqueous phase. The average diameter of the oil droplets in these HIPEs were around 10–25 μm.

The microscopy images indicated that the coconut oil HIPEs had a very different structure than the soybean oil ones. They appeared to consist of both spherical and irregular shaped protein-rich aqueous domains dispersed within a continuous oil phase. These results suggest that these HIPEs had undergo a transition from an oil-in-water to a water-in-oil structure during their formation. This kind of phase inversion can be promoted by the presence of a partly crystalline oil phase in an oil-in-water emulsion due to partial coalescence [27]. When the lipid phase inside an oil droplet is partly crystalline, a fat crystal from one droplet can penetrate into the liquid region of another droplet, which leads to the formation of a crosslink. If this process continues, then the fat droplets can form large clusters, eventually leading to phase inversion. The overall rheological properties of emulsions are typically dominated by those of the continuous phase. Consequently, in the coconut oil HIPEs, one would expect them to be dominated by the rheology of the partially crystalline oil phase.

Overall, these experiments indicate that the soybean oil HIPEs had a microstructure closer to that of real beef adipose tissue, whereas the coconut oil HIPEs had an appearance and texture that was closer. Thus, mimicking the microstructure of real adipose tissue does not necessarily lead to the required physicochemical and functional attributes.

#### 3.2.2. Temperature Dependence of Rheological Properties

**Figure 7:**
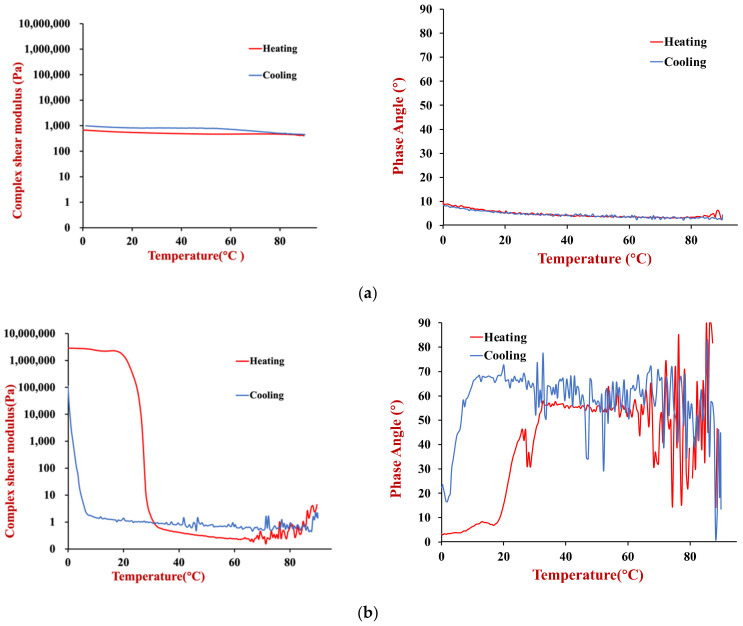
(**a**) Change in complex shear modulus and phase angle with temperature of soybean oil HIPEs during heating and cooling. (**b**) Change in complex shear modulus and phase angle with temperature of coconut oil HIPEs during heating and cooling.

The temperature dependence of the rheological properties of the HIPEs prepared using the two oils (soybean or coconut oil) were measured. The complex shear modulus and phase angle of the samples were measured when they were heated and cooled in a controlled manner (Figure 7).

The soybean oil HIPEs had a relatively low shear modulus and phase angle across the whole temperature range during both heating and cooling (Figure 7a). These results suggest that they were relatively soft materials that were predominantly elastic, rather than viscous. Moreover, there was no evidence of any distinct thermal transitions across the temperature range studied. Presumably, this is because the soybean oil remained liquid across the whole temperature range studied and because the soy proteins used as emulsifiers were already denatured prior to utilization.

The coconut oil HIPEs exhibited very different thermal characteristics to the soybean oil ones (Figure 7b). Initially, we consider the changes that occurred when they were heated. From 0–30 °C, these HIPEs had a relatively high shear modulus, which can be attributed to the mechanical rigidity resulting from the formation of a 3D crystal network by the coconut oil. From 30–35 °C, there was a sharp decrease in the shear modulus of the HIPEs, which was due to melting of the coconut fat and disruption of this network. From 35–90 °C, the shear modulus remained relatively low with increasing temperature, which indicates that the emulsions were relatively soft. When the same samples were cooled, they the shear modulus remained relatively low from 90 to 5 °C, but then increased steeply, which was attributed to fat crystallization. The crystallization temperature was considerably lower than the melting temperature due to supercooling effects [27] Initially, the phase angle of the coconut oil HIPEs was relatively low (<25°), which indicated that they were predominantly solid-like. However, the phase angle exceeded 45° when the samples were heated above about 35 °C, which indicated they became more fluid-like.

#### 3.2.3. Differential Scanning Calorimetry

**Figure 8:**
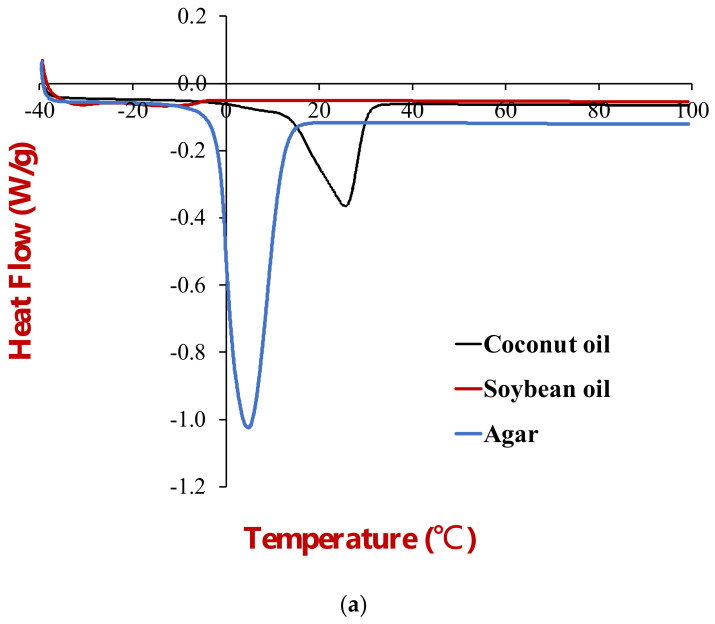

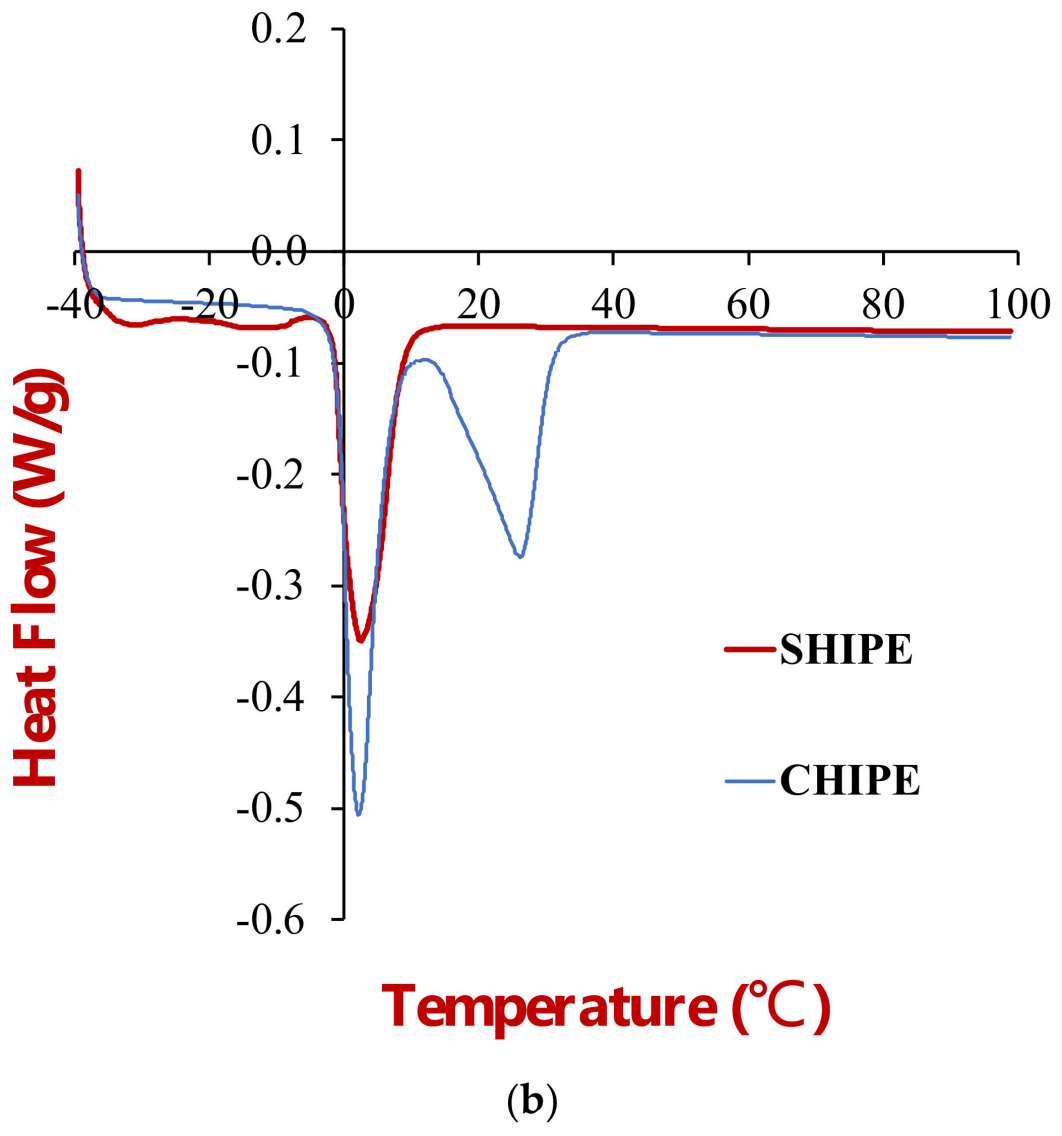
(**a**) DSC profiles of coconut oil, soybean oil, and agar when they were heated from −40 to 100 °C. (**b**) DSC profiles of coconut oil HIPES (CHIPE) and soybean oil HIPEs (SHIPES).

DSC was used to characterize the thermal behavior of the individual ingredients (Figure 8a) and the emulsions (Figure 8b) by measuring the change in heat flow versus temperature.

The soybean oil exhibited no major peaks when the samples were heated from −40 to 100 °C, which suggests that it remained predominantly liquid across the whole temperature range studied. However, there were some broad minor endothermic peaks from −40 to 0 °C, which suggests that a small fraction of the soybean oil may have been in a crystalline form. The thermal behavior of the soybean oil can be attributed to the fact that it contains relatively low levels of long-chain saturated fatty acids, which have high melting points. Soybean oil has been reported to contain only around 11% palmitic acid and 4% stearic acid [28]. A fraction of these saturated fatty acids may have crystallized when the samples were frozen, but the majority of the unsaturated fatty acids remained in a liquid form.

The DSC profiles for the bulk coconut oil showed that it melted over a temperature range from 13 to 33 °C, with an endothermic peak around 26 °C. Coconut oil has been reported to contain around 49% lauric acid, 8% caprylic acid, 8% myristic acid, and 8% capric acid, with an overall saturated fat content exceeding 72% [29]. This high level of saturated fatty acids leads to a relatively high melting point because the linear chains can pack closely together.

The DSC profile of an agar solution (2%) was also tested because it was used in the aqueous phase of the gelled emulsions (discussed in Section 3.3). We also characterized the thermal behavior of a soybean protein solution (data now shown). It exhibited a larger endothermic peak just above 0 °C, which was attributed to melting of ice crystals in the aqueous solution. No peaks were observed at higher temperatures, which was attributed to the fact that the proteins were already denatured prior to utilization.

The thermal behavior of the soybean and coconut oil HIPEs was also characterized using DSC (Figure 8b). The thermal peaks of the HIPEs were consistent with those of the main ingredients they contained. The soybean oil HIPEs exhibited some minor endothermic peaks from −40 to 0 °C, which overlapped with those seen in the bulk soybean oil (Figure 8a). The larger endothermic peak at 0 °C was consistent with the melting of ice crystals observed in the protein solutions No peaks were observed at higher temperatures because the soybean oil was already in a liquid state. In contrast, the coconut oil HIPEs exhibited two major endothermic peaks, one around 0 °C due to ice crystal melting and one around 20–30 °C due to coconut fat crystals melting.

#### 3.2.4. Heating and Freezing Stability

**Figure 9:**
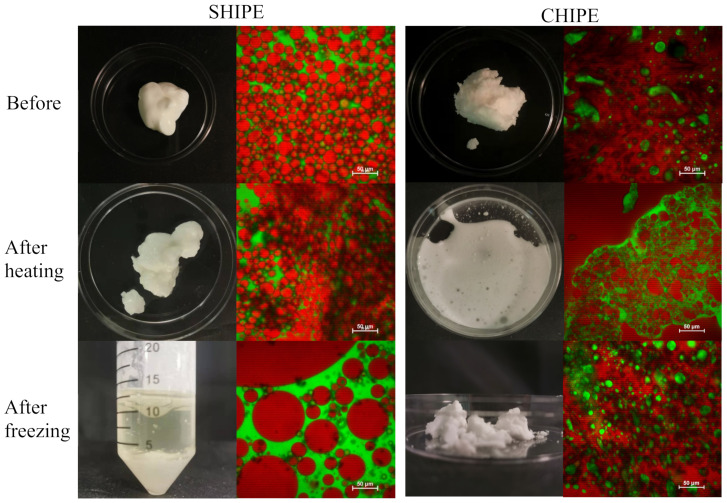
Appearance and microstructure of HIPEs containing soybean oil or coconut oil before and after heating for 30 min or freezing for 24 h and then thawing. The oil is stained red and the proteins green in the microscopy images.

The heating and freezing stability of the two HIPEs was characterized to ascertain whether their thermal behavior was similar to that of beef adipose tissue (Section 3.1.4). Visually, the soybean oil HIPEs looked similar before and after heating (Figure 9). However, the microscopy images showed that some aggregation of the oil droplets had occurred during heating, which may have been due to an increase in the hydrophobic attraction between the protein-coated oil droplets at higher temperatures. The freezing and thawing of the soybean oil HIPEs had a much bigger impact on their appearance and microstructure. After thawing, these emulsions separated into two layers: a cloudy aqueous lower layer and a clear oily upper layer. The volume ratio of the upper and lower layers was similar to the oil-to-water ratio in the original emulsion. The microscopy images showed that extensive coalescence had occurred in the soybean oil HIPEs, with the individual oil droplets being much larger than those in the original emulsion. These results suggest that extensive droplet coalescence and oiling off occurred during the freezing and thawing process. This effect can be attributed to the fact that the oil droplets are forced together when the water phase crystallizes, which disrupts the protective coating around them [30]. Consequently, when they are subsequently thawed the oil droplets merge together and separate.

The coconut oil HIPEs behaved very differently to the soybean oil ones when they were frozen or heated (Figure 9). Visual observations indicated that these HIPEs broken down when they were heated, leading to the formation of an opaque whitish fluid. The microscopy images showed that some of the oil had formed a separate phase after heating. Interestingly, this separated oil phase was surrounded by regions consisting of oil droplets suspended in water, which indicates that a partial phase inversion from a water-in-oil to an oil-in-water emulsion occurred during the heating and cooling process. Presumably, the 3D crystal network in the continuous phase of the water-in-oil emulsions disintegrated during heating as the coconut fat crystals melted.

The coconut oil HIPEs were more resistant to freezing than the soybean oil ones. Visually, they were still whitish semi-solid materials after freezing and thawing, although they appeared to be less sticky after freezing. The microscopy images showed that these emulsions retained their water-in-oil structure after freezing and thawing, with small water droplets being dispersed in a fatty continuous phase. The good freeze–thaw stability of these systems may have been because the fat phase crystallized and formed a 3D network that trapped the water droplets inside so they could not move and interact with each other. As a result, there was little droplet aggregation in these emulsions. The higher solid fat content of the coconut oil HIPEs after freezing made them more brittle, so they tended to break into smaller pieces when handled.

In summary, these results show that the thermal stability of the HIPEs is highly dependent on the nature of the oil phase used to formulate them. Soybean oil HIPEs are more resistant to heating, whereas coconut oil HIPEs are more resistant to freezing.

### 3.3. Gelled Emulsions

As discussed earlier, the freezing and heating behavior of the two kinds of HIPEs were different from those of the beef adipose tissue. For this reason, we also characterized the thermal behavior of gelled emulsions formulated from soybean oil and coconut oil. These gelled emulsions contained a cold-set gelling polysaccharide (agar) to modulate their freezing and heating behavior. Only 60% oil could be incorporated into these emulsions (rather than the 75% oil in the HIPEs) because the presence of the agar greatly increased the viscosity of the aqueous phase, which reduced the mixing efficiency at higher oil levels.

#### 3.3.1. Appearance and Microstructure

**Figure 10:**
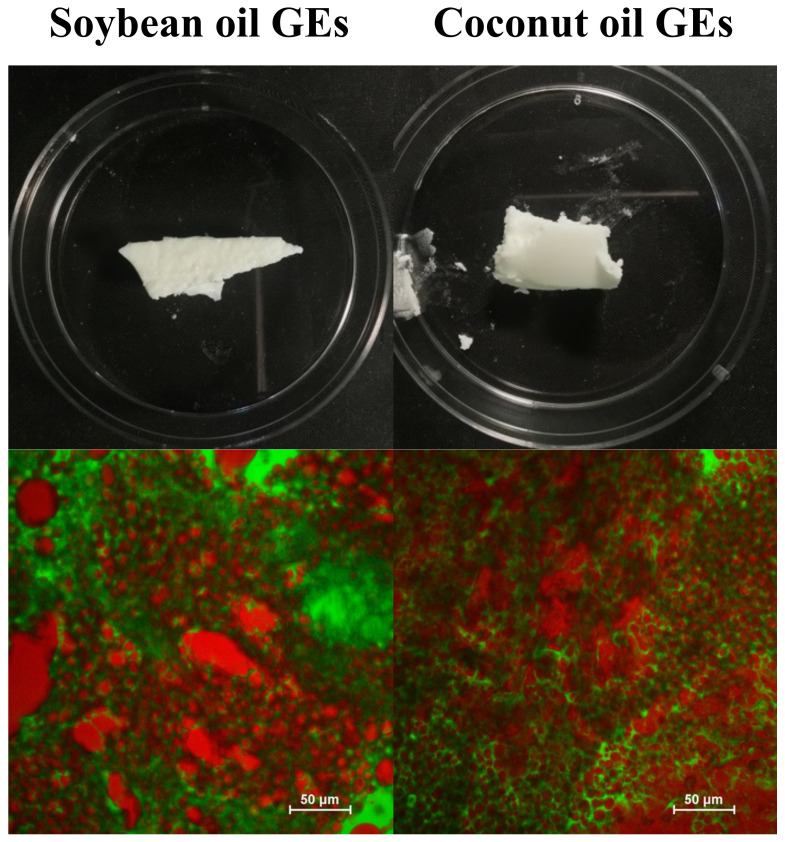
Appearance and microstructure of soybean oil gelled emulsions and coconut oil gelled emulsions. The fat phase is stained red and the proteins are stained green.

Both types of gelled emulsions appeared to be whitish opaque semi-solid materials (Figure 10). The microstructures of these emulsions were also fairly similar, consisting of fat droplets with a broad range of sizes and shapes dispersed in a protein-rich aqueous phase. Both the soybean oil gelled emulsions and HIPEs were of the oil-in-water type. These results suggest that the lower oil droplet concentration in the gelled emulsions, as well as the presence of the agar in the aqueous phase, may have inhibited the coalescence and phase inversion of the oil droplets. A highly viscous or gelled aqueous phase would have retarded oil droplet movement, thereby preventing them from coming close enough to coalesce.

The microscopy images showed that some of the oil droplets formed large irregular clusters in both types of gelled emulsions, however, there was also evidence of some individual oil droplets. The clustering of the oil droplets in these systems may have been due to some depletion flocculation caused by the presence of non-adsorbed agar in the aqueous phase [31]. Non-adsorbed polysaccharides are excluded from a narrow region around the surface of each oil droplet, which generates an osmotic pressure that forces them together. At the relatively high temperatures used during emulsion preparation, which were above the gelation temperature of the agar, the oil droplets could move through the aqueous phase and interact with each other. However, once the emulsions were cooled and the agar gelled, the oil droplets would have been held in place due to the mechanical rigidity of the biopolymer network.

#### 3.3.2. Temperature Dependence of Rheological Properties

**Figure 11:**
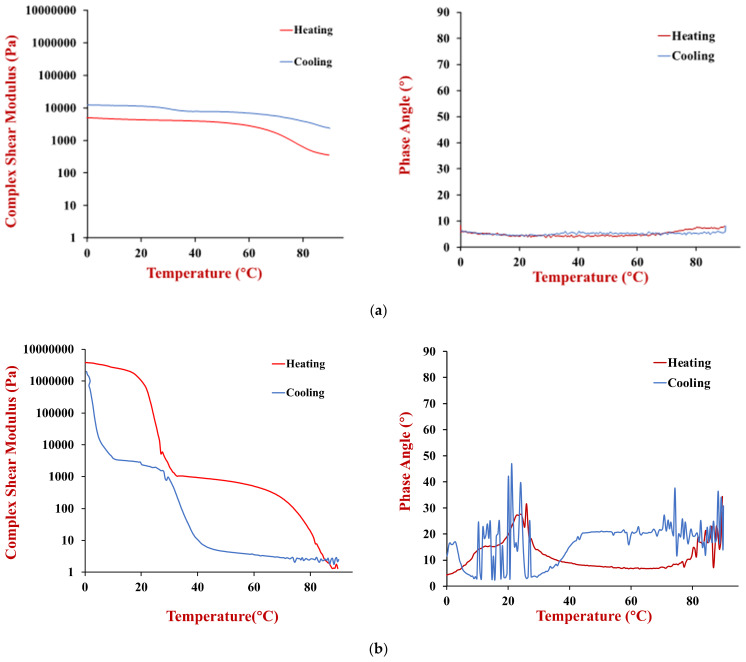
(**a**) Complex shear modulus and phase angle of soybean oil gelled emulsions during heating and cooling. (**b**) Complex shear modulus and phase angle of coconut oil gelled emulsions during heating and cooling.

The dynamic shear rheology of the gelled emulsions and the agar solution used to prepare them were measured as a function of temperature to characterize their thermal gelation properties (Figure 11).

For the soybean oil emulsions (Figure 11a), the gelled emulsions had a much higher complex shear modulus (4310 Pa) than the HIPEs (542 Pa) at 20 °C, which can be attributed to the mechanical rigidity provided by the agar hydrogel in the aqueous phase of the gelled emulsions. The much higher shear modulus of the emulsion gels than the HIPEs persisted as the samples were heated to around 70–80 °C because the agar remained in a gelled state over this temperature range. However, there was a pronounced decrease in the shear modulus of the emulsion gels when they were heated to higher temperatures, which can be attributed to a gel-to-sol transition of the agar. The shear modulus of the soybean oil emulsion gels remained relatively constant when they were cooled, which can be attributed to the fact that the agar gels at a much lower temperature than it melts. The fact that the shear modulus was higher during cooling than heating may have been due to some evaporation of water when the samples were held at the higher temperatures, or due to aggregation of the protein molecules and protein-coated oil droplets. The phase angle remained between 5 to 10° during both heating and cooling, which suggests that the emulsion gels were always predominantly elastic-like materials. This may have been because of the strong droplet aggregation promoted by depletion flocculation discussed earlier.

The coconut oil gelled emulsions (Figure 11b) exhibited very different rheological behavior during heating and cooling than the soybean oil ones (Figure 11a) or the coconut oil HIPEs (Figure 7b). During heating, the coconut oil gelled emulsions exhibited two distinct melting stages. First, there was a pronounced reduction in the complex shear modulus around 20–30 °C, which can be attributed to melting of the coconut fat crystals. Interestingly, the emulsion gels had an oil-in-water structure, suggesting that changes in the solid fat content of the lipid phase altered the interactions between the oil droplets. Studies have shown that fat crystals can penetrate from one droplet to another in oil-in-water emulsions with partially crystalline oil phases, which could increase their solidity [32]. The coconut oil gelled emulsions exhibited a second steep reduction in their shear modulus around 70–80 °C, which can be attributed to the gel-to-sol transition of the agar around this temperature. Over the temperature range studied, the magnitude of the decrease in the shear modulus of these emulsion gels was fairly similar to that of real adipose tissue (Figure 3).

During cooling, there were also two distinct changes in the rheological properties of the coconut oil emulsion gels, but these occurred at lower temperatures than during heating. There was a steep rise in the shear modulus around 40–30 °C, which can be attributed to a sol-to-gel transition of the agar and another steep rise around 10–0 °C, which can be attributed to crystallization of the coconut oil. The reason these transitions occur at a lower temperature during cooling than during heating, can be attributed to supercooling and thermal hysteresis effects.

The phase angle results suggested that the coconut oil emulsion gels remained predominantly elastic throughout the heating and cooling process. However, there were some distinct fluctuations in their values, especially during cooling. There appeared to be peaks in the phase angle at temperatures where phase transitions were occurring, suggesting the samples became somewhat more fluid-like in these regions.

#### 3.3.3. Differential Scanning Calorimetry

**Figure 12:**
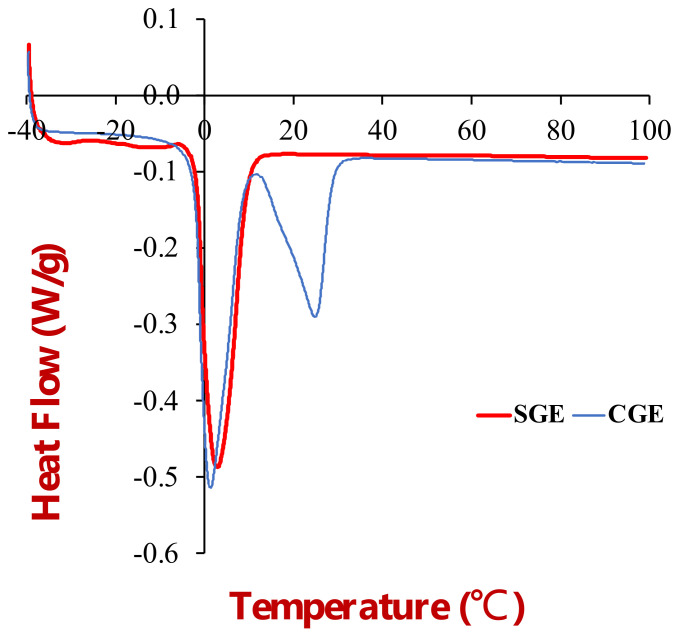
DSC profiles of soybean oil (SGE) and coconut oil (CGE) emulsion gels during heating from −40 °C to 100 °C.

The DSC profiles of the two gelled emulsions were measured when they were heated at a controlled rate (Figure 12). For both gelled emulsions, there was a large endothermic peak just above 0 °C, which was attributed to the melting of ice crystals formed when the samples were frozen. For the coconut oil gelled emulsions there was another large endothermic peak around 25 °C, which was attributed to the melting of the coconut fat crystals. No thermal transitions were observed at higher temperatures. A DSC analysis of the 2% agar solution used to prepare the gelled emulsions also only showed an endothermic transition corresponding to the melting of ice crystallization. Presumably, the enthalpy change associated with the melting of the agar gel was not high enough to be detected by the calorimeter used in this study.

#### 3.3.4. Heating and Freezing Stability

**Figure 13:**
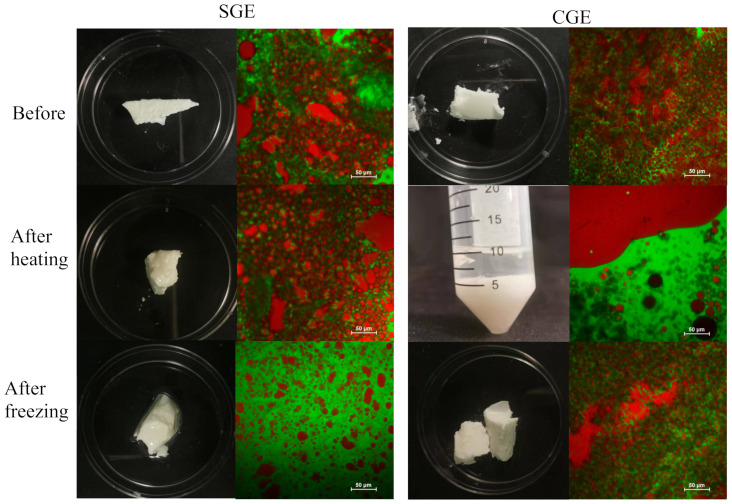
Appearance and confocal microscope images of coconut oil and soybean oil gelled emulsions (GEs) before and after heating and freezing.

The heating and freezing stability of the two gelled emulsions was also examined to ascertain their ability to mimic the thermal stability of real beef adipose tissue (Figure 13). After heating, the soybean oil gelled emulsions maintained their semi-solid characterize and opaque whitish appearance and had shiny surfaces, which was similar to observed for the soybean oil HIPEs. The shiny surface appearance of these samples was related to the fact that they were not hard solids, so some flow of the emulsion could occur, leading to a smooth surface that gave specular reflectance of light wavelengths. The microstructure of the soybean oil gelled emulsions was relatively similar before and after heating, consisting of a mixture of individual droplets and irregular shaped droplet aggregates dispersed in a protein-rich aqueous phase. There was some visible phase separation of the soybean oil gelled emulsions after freezing, with evidence of some oiling off and some gelled emulsion remaining. The microstructure of these samples showed that the whitish portion consisted of an oil-in-water emulsion. Notably, the oil droplets remaining appeared to be smaller and fewer in number than in the original samples before freezing. This suggests that some of the larger individual droplets and droplet aggregates in these gelled emulsions may have coalesced during the freezing and thawing process, leaving the smaller ones behind.

The coconut oil gelled emulsions exhibited an opposite effect to the soybean oil ones. There were unstable to heating but stable to freezing, which is similar to observed for the coconut oil HIPEs. These results indicate that the nature of the oil phase used to formulate gelled emulsions has a profound influence on their thermal stability, which may be important for their application as adipose tissue substitutes in plant-based food products.

### 3.4. Texture Profile Analysis

The textural characteristics of selected plant-based adipose tissue analogs was tested and compared to that of real adipose tissue by performing texture profile analysis (TPA). TPA compresses a sample twice to mimic the behavior of foods within the human mouth during mastication. The force versus time profiles of each sample were recorded by the instrument and used to calculate their TPA parameters (Table 1).

The real adipose tissue had a much higher hardness value than any of the plant-based adipose tissue analogs. The TPA measurements were carried out at ambient temperature. Under these conditions, the beef fat and coconut oil would be expected to be partially crystalline, whereas the soybean oil would be liquid. However, the beef fat should have a higher solid fat content than the coconut oil at this temperature because it has a higher melting point. Among the plant-based adipose tissue samples, the hardness decreased in the following order: CGE > CHIPE ≈ SGE > SHIPE. The results suggest that coconut oil is more suitable for mimicking the hardness of beef adipose tissue than soybean oil, and that gelled emulsions are more suitable than HIPEs. The highest hardness for the coconut oil gelled emulsions can be attributed to the combined effects of the agar gel and fat crystal network on the mechanical rigidity of the samples. The chewiness parameters exhibited a similar trend as the hardness. All the plant-based adipose tissue analogs had a lower adhesiveness than beef adipose tissue, suggesting there were some differences in the nature of the molecular interactions occurring at the surfaces of the different samples. Beef adipose tissue contains connective tissue, which may have increased its stickiness.

In general, none of the plant-based adipose tissue had TPA parameters that were similar to the beef adipose tissue. However, the gelled emulsions formulated with the coconut oil were the closest. They were relatively hard, melted during heating, solidified during cooling, and had an appearance like real beef adipose tissue. Consequently, they may be suitable for application in some kinds of plant-based meat and seafood analogs. However, these gelled emulsions were unstable to heating, which may limit their application in many products.

### 3.5. Summary of Proposed Mechanisms

**Figure 14:**
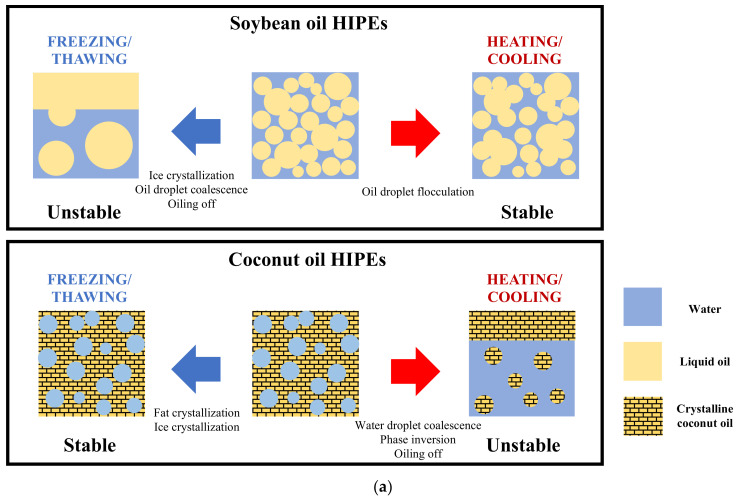

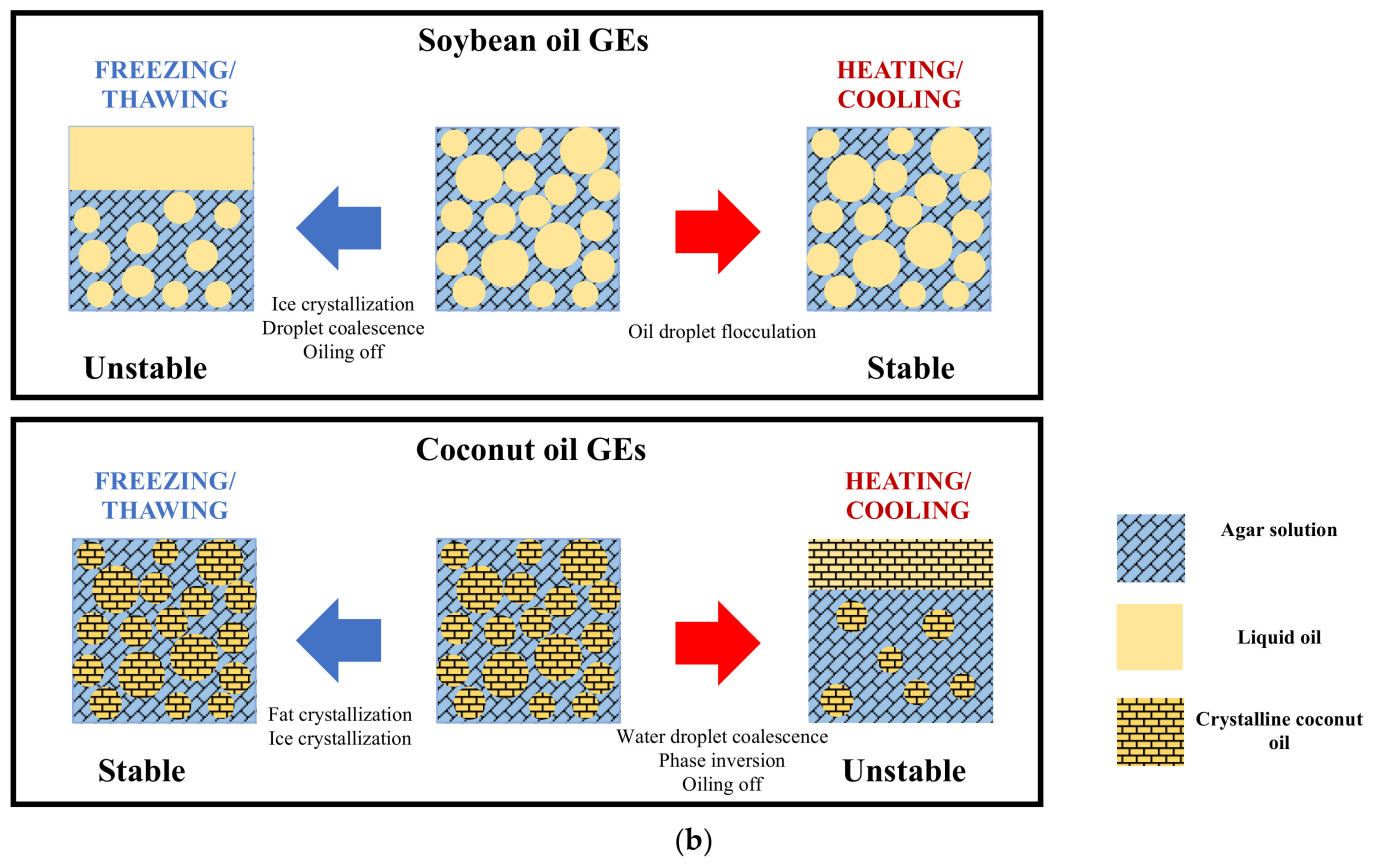
(**a**) Schematic diagram of the behavior of soybean and coconut oil HIPEs after freezing and heating. (**b**) Schematic diagram of the behavior of soybean and coconut oil gelled emulsions after freezing and heating.

Finally, we provide a schematic of the key physicochemical processes that are believed to occur inside the different kinds of emulsions during heating and freezing (Figure 14).

SHIPEs: At room temperature, the soybean oil HIPEs are concentrated oil-in-water emulsions (Figure 14a). After heating and then cooling back to room temperature, the SHIPEs undergo a limited amount of droplet flocculation but remain as opaque white semi-solid materials that have properties somewhat like beef adipose tissue. After freezing and then thawing, the SHIPEs breakdown, exhibiting coalescence and oiling off of the oil droplets. These effects can be attributed to the fact that the oil droplets are forced close together when the water crystallizes.

CHIPEs: At room temperature, the coconut oil HIPEs are water-in-oil emulsions containing a semi-crystalline continuous phase consisting of aggregated coconut oil fat crystals (Figure 14a). After heating and cooling back to room temperature, the CHIPEs undergo a phase inversion and form an oil-in-water emulsion in contact with a separated oil phase. Given sufficient time, the oil phases in these emulsions crystallize. After freezing and thawing, the CHIPEs remain relatively stable because the fat crystal network stops the water droplets from coming into contact.

SGEs: At room temperature, the soybean oil gelled emulsions are concentrated oil-in-water emulsions consisting of liquid oil droplets embedded in an agar hydrogel (Figure 14b). After heating and cooling back to room temperature, the SGEs remain relatively stable because the agar in the aqueous phase prevents the oil droplets from coming into contact and aggregating. After freezing and thawing, the SGEs partially breakdown, and form two phases: an oil phase and an oil-in-water emulsion phase. Presumably, some of the larger oil droplets coalesce when the emulsions are frozen and ice crystals form. Even so, a whitish opaque semi-solid material was still present after freezing, so they retained some of the characteristics of adipose tissue.

CGEs: At room temperature, the coconut oil gelled emulsions are oil-in-water emulsions, consisting of partially crystalline oil droplets dispersed within an agar hydrogel (Figure 14b). After heating and cooling back to room temperature, the CGEs separate into two phases: an oil phase and a dilute oil-in-water emulsion. This result suggests that some phase inversion occurred during heating, which may have been initiated by partial coalescence of the coconut oil droplets. A fat crystal from one oil droplet penetrated the liquid portion of another oil droplet, leading to the formation of clumps. During heating, the coconut oil completely melted and the oil droplets in the clumps merged and formed a separate phase. Presumably, the larger oil droplets were more prone to this effect, leaving a dilute oil-in-water emulsion phase containing small oil droplets. After freezing and thawing, the CGEs remained relatively stable because the agar hydrogen prevented the water droplets from coming into contact, and the fully crystalline fat droplets could not merge. The coconut oil gelled emulsions therefore retained much of the characteristics of beef adipose tissue after freezing.

## 4. Conclusions

In this study, the microstructure, texture, and thermal stability of beef adipose tissue were compared to plant-based adipose tissue analogs formulated from either HIPEs or emulsion gels. The impact of the melting characteristics of the lipid phase was also examined by using a low melting point (soybean oil) and high melting point (coconut oil) fat source. Beef adipose tissue was a whitish opaque semi-solid that was relatively resistant to freezing and heating because of its cellular structure. At room temperature, the beef adipose tissue was relatively hard and underwent considerably softening when heated above about 35 °C. All HIPEs and gelled emulsions were also whitish opaque semi-solids and so could mimic some of the desirable attributes of adipose tissue. However, their hardness and melting/crystallization characteristics were quite different from adipose tissue. The HIPEs and gelled emulsions containing coconut oil were most able to mimic the properties of the beef adipose tissue in terms of hardness and melting behavior. However, these emulsions were unstable to heating, undergoing phase separation and oiling off, which would be unsuitable for many cooked meat analogs. The soybean oil HIPEs and gelled emulsions had better heat stability, but they tended to breakdown after freezing. Overall, this study provides some valuable insights into the formulation of plant-based alternatives to animal fats. In future studies, it will be important to assess the behavior of these plant-based adipose tissue analogs when they are incorporated into meat or seafood analogs. For instance, can they be used to simulate the marbling appearance of beef tissue or to create subcutaneous fat layer in pork products.

## Figures and Tables

**Table 1:** Texture profile analysis of beef adipose tissue, soybean oil HIPEs (SHIPE), coconut oil HIPES (CHIPE), soybean oil gelled emulsions (SGE), and coconut oil gelled emulsions (CGE).

Parameter	Beef Adipose	SHIPE	CHIPE	SGE	CGE
Hardness (g)	23,300 ± 2900 ^c^	14.79 ± 0.59 ^a^	1050 ± 240 ^ab^	1420 ± 470 ^ab^	2262 ± 99 ^b^
Adhesiveness (g/s)	−516 ± 470	−101 ± 45	−77 ± 10	2.8 ± 5.0	−47 ± 28
Resilience (%)	21 ± 17 ^ab^	13.9 ± 4.2 ^ab^	0.11 ± 0.04 ^a^	55.2 ± 4.2 ^c^	28.9 ± 1.5 ^b^
Cohesion	0.53 ± 0.11	0.36 ± 0.09	0.35 ± 0.11	0.92 ± 0.03	1.8 ± 1.5
Springiness (%)	57 ± 44	81 ± 13	91.7 ± 9.0	96.9 ± 1.0	91.9 ± 5.2
Chewiness	10,000 ± 4600 ^b^	4.3 ± 1.2 ^a^	322 ± 74 ^a^	1280 ± 440 ^a^	2290 ± 500 ^a^

Data denoted with ^a b c^ are significantly different (*p* < 0.05).

## Data Availability

All related data and methods are presented in this paper. Additional inquiries should be addressed to the corresponding author.
